# Oral Administration of Heat-Treated Lactobacilli Modifies the Murine Microbiome and Reduces *Citrobacter* Induced Colitis

**DOI:** 10.3389/fmicb.2020.00069

**Published:** 2020-01-30

**Authors:** Alicja K. Warda, Pedro H. de Almeida Bettio, Cara M. Hueston, Giulio Di Benedetto, Adam G. Clooney, Colin Hill

**Affiliations:** ^1^APC Microbiome Ireland, University College Cork, Cork, Ireland; ^2^School of Microbiology, University College Cork, Cork, Ireland

**Keywords:** microbiome, dead probiotics, inflammation, pharmabiotics, *Citrobacter*

## Abstract

Significant evidence supports a relationship between the gut microbiome, inflammation, host response, and health, including the finding that a number of disorders are associated with disruption of the microbiome. In these disorders, a number of dietary interventions (including prebiotics, live probiotics, or heat-killed microbes) have been proposed to be curative or preventative agents. The use of heat-killed microbes has a number of benefits over living organisms, including reduced infection risk in vulnerable individuals, extended shelf life and the potential for use in combination with antimicrobial agents. We previously reported that murine chow supplemented with 5% ADR-159, a heat-treated fermentate generated by two *Lactobacillus* strains, altered both behavior and the microbiome of male mice. Now we show that ADR-159 fed female mice also display a similar microbiome shift as determined by 16S rDNA analysis. In particular, we observed a reduction of levels of *Turicibacter* and *Clostridium sensu stricto*. These subtle changes in the bacterial component of the microbiome were mirrored by changes in the virome. Extended consumption of the ADR-159 diet had no negative effect on general health and lipocalin 2 levels (LCN2; a proxy for inflammation), but we observed increased IL-17f and decreased IL-12α expression in the colon and decreased short chain fatty acid levels in the ADR-159 fed animals. Four weeks into the diet, half of the animals were dosed with *Citrobacter* to determine the effect of ADR-159 on infection and on pathogen induced colitis. Overall, our results suggest that while the ADR-159 diet does not prevent *Citrobacter* infection, it had an effect on *Citrobacter*-induced inflammation. In contrast to animals fed standard chow, ADR-159 fed animals did not show a reduction of small intestine length and increase of colon crypt depth, which occurred in control mice. These microbiological, histological, and immunological results provide evidence to support the impact of heat-treated microorganisms and their metabolites on the murine microbiome and health.

## Introduction

Significant evidence supports the relationship between the gut microbiome, inflammation, host response, and health (Pedersen et al., 2016; Dinan and Cryan, 2017; Kelly et al., 2017; Giron and Quigley, 2018). Disruption of the microbiome can lead to changes in microbial metabolism and consequently host metabolism (Belizario et al., 2018). The aforementioned changes can also affect inflammatory responses and adaptive immunity and can even contribute to metabolic disorders (Belizario et al., 2018). Inflammation is part of a multifaceted biological response to pathogens, damaged cells or irritants (Chen et al., 2018). Inflammatory processes can clear necrotic cells and damaged tissues and initiate tissue repair (Ferrero-Miliani et al., 2007). While acute inflammation in healthy individuals is considered an indication of healing, it can cause a major disturbance when it is inappropriate or becomes chronic. For example, Inflammatory Bowel Disease (IBD) is a chronic inflammatory condition affecting the gastrointestinal (GI) tract, and includes Crohn’s Disease (CD) and ulcerative colitis (UC) (Rubin et al., 2012). Normally, dead or damaged cells in the gastrointestinal tract are rapidly eliminated while new ones are constantly generated (Kim et al., 2010). A typical cell turnover of 4–5 days is associated with a basal level of inflammation that facilitates maintenance of the intestinal barrier and prevents an excessive inflammatory response (Kim et al., 2010). In the absence of the microbiome, cell turnover is slowed; one of the effects of this is an excessive inflammatory response (Kim et al., 2010). During bacterial infection the balance of cell renewal can be disturbed, resulting in rapid proliferation and crypt hyperplasia (Kim et al., 2010). This phenomenon is commonly observed in animal models of GI infection and inflammation. A frequent cause of bacterial infection and intestinal inflammation include members of the Proteobacteria; especially food-born *Campylobacter*, *Salmonella*, *Shigella*, enteroinvasive and enterohemorrhagic *Escherichia coli* (EIEC and EHEC, respectively), and *Yersinia* species (Papaconstantinou and Thomas, 2007). In murine models the presence of certain bacteria, including *Proteus mirabilis*, *Klebsiella pneumonia*, and *Bilophila wadsworthia* has been associated with colitis (Weingarden and Vaughn, 2017). Bacteria such as *Citrobacter rodentium*, *Helicobacter pylori*, *Mycobacterium avium*, and *Salmonella enterica* serovar Typhimurium are commonly used in animal models to trigger colitis-like conditions (Jiminez et al., 2015). In particular, the *C. rodentium* infection is used as a model for several important human intestinal disorders, including CD, UC, and colon tumorigenesis (Collins et al., 2014b). Infection of mice with *C*. *rodentium* results in the colonization of the large intestine and subsequent mild inflammation that is maintained after the pathogen has been cleared (Wiles et al., 2004).

*Citrobacter* has been associated with a T helper 1 (Th1) and a more potent Th17 cell response (Higgins et al., 1999; Mangan et al., 2006; Collins et al., 2014b). A number of Interleukins (IL-) and cells create an interconnection between the innate and adaptive immune systems, allowing the host to overcome the infection. The Th1 cell response in the gut is characterized by the production of interferon-γ (IFN-γ) and tumor necrosis factor (TNF) (Collins et al., 2014b). TNF is both a pro- and anti-inflammatory cytokine involved in the development of autoimmune diseases and cancer, as well as playing a role in protection against infectious pathogens (Mehta et al., 2018). The Th17 cell response is characterized by the production of IL-17a, IL-17f, IL-22, and IL-26 (Li et al., 2018). A robust expression of pro-inflammatory cytokines (IL-17a, IL-17f, IL-22, and IFN-γ) peaking at day 8 and subsequently declining at day 11 were reported for *Citrobacter* infection (Hoffmann et al., 2009). At the same time, IL-6 has been reported to provide protection against the colonic mucosal ulceration caused by *Citrobacter* (Dann et al., 2008), while IL-1β has been shown to contribute to *Citrobacter* clearance and minimize tissue damage (Alipour et al., 2013). *C*. *rodentium* is a known attaching and effacing (A/E) pathogen that induces a coordinated response involving multiple components of the mucosal defense system (Collins et al., 2014b).

The potential of heat-killed microorganisms to provide a benefit to humans or animals has been examined in a number of studies (Taverniti and Guglielmetti, 2011; de Almada et al., 2016; Warda et al., 2018). These heat-killed preparations have also been termed “paraprobiotics,” “post-biotics,” or “ghost probiotics” (Taverniti and Guglielmetti, 2011; de Almada et al., 2016). Such preparations containing heat-killed microorganisms have been reported to confer health benefits including treatment of diarrhea, colitis, inflammation, respiratory diseases, intestinal lesions, as well as modification of the immune system and impact on the intestinal microbiome and bacterial translocation (de Almada et al., 2016). The administration of microorganisms that have been subjected to a high temperature heat treatment could be advantageous over living microbes in terms of (i) extended shelf life, (ii) not being affected by high or low temperature excursions (allowing for product to be more readily sanitized or even sterilized), (iii) storage and transportation at ambient temperatures, with a consequent potential for uses in less developed regions. Additional advantages include (iv) limited infection risk in vulnerable individuals, (v) no translocation of genetic material for those preparations subjected to a sufficient heating step, and (vi) the ability to be used in combination with antimicrobial agents (Taverniti and Guglielmetti, 2011; de Almada et al., 2016; Warda et al., 2018).

ADR-159 contains a heat-treated fermentate and microbial biomass generated by two *Lactobacillus* strains, *Lactobacillus fermentum*, and *Lactobacillus delbrueckii*. In previous work, we have demonstrated that an ADR-159 supplemented diet is capable of modifying the microbiome and behavior in healthy male mice (Warda et al., 2018). We used the *C. rodentium* model of infection and colitis to elucidate the role of ADR-159 in infection and inflammation.

## Materials and Methods

### Diet Preparation

The composition of the ADR-159 preparation has been previously described as follows (Warda et al., 2018): ADR-159 contains a co-fermentate of *Lactobacillus fermentum* and *Lactobacillus delbrueckii*, which also includes culture medium (lactose monohydrate, casein peptone, yeast extract, sodium acetate, dipotassium phosphate). The fermentate is subjected to an extended high-temperature treatment post-production. One gram of ADR-159 contains a minimum of 60 billion intact bacterial cells. ADR-159 is a proprietary product produced and supplied by Adare Pharmaceuticals SAS, Route de Bu, 78550, Houdan, France. ADR-159 was incorporated into standard mice chow [2018S Teklad Global 18% Protein Rodent Diet (Envigo)] to a final concentration of 5%, equivalent to approximately 3 × 10^9^ cell bodies per gram of chow.

### Animal Experiment

#### Animals and Housing Conditions

**FIGURE 1:**
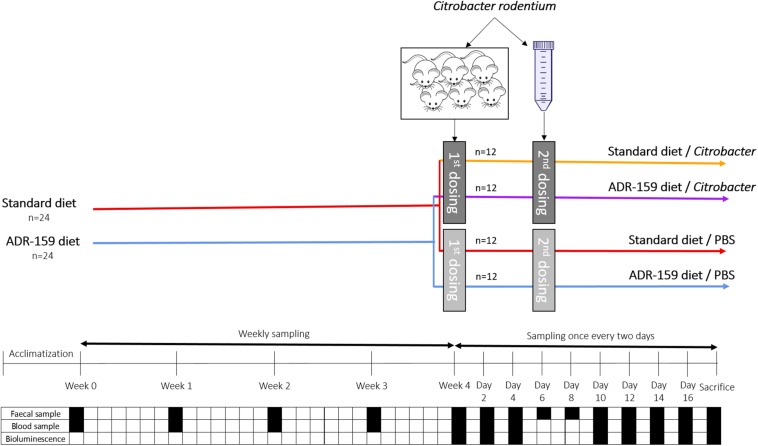
Schematic representation of *C*. *rodentium* animal experiment. Dark gray indicates dosing containing *Citobacter* while light gray indicated dosing of animals with PBS. Black square indicates sampling, while white square indicates no sampling of given material type.

Animals and housing conditions used were similar to those previously reported (Warda et al., 2018) with the following modifications. Briefly, 54 eight-week old female C57BL/6 mice (Envigo, United Kingdom) were randomly assigned in groups of 3 or 4 animals to 14 enriched (cardboard tubes and shredded paper) individually ventilated cages for 1 week of acclimatization at 21 ± 1°C and at a humidity of 55 ± 10%, and with a 12 h light/dark cycle. Animals were fed *ad libitum* with 2018S Teklad Global 18% Protein Rodent Diet (Envigo; 8 cages/30 animals) or the same diet supplemented with 5% ADR-159 (6 cages/24 animals) (Figure 1). Each week animals were weighed and feed consumption was controlled by weighing contents of the food hoppers. Additionally, fecal samples were acquired for 16S rRNA gene metagenomics, phageomics and lipocalin analysis.

All experiments were performed in accordance with the European Directive 2010/63/EU and approved by the Animal Experimentation Ethics Committee of University College Cork and Health Products Regulatory Authority under HPRA Project License AE19130/P060. All efforts were made to reduce the number of animals and to minimize suffering.

#### *Citrobacter* Infection

*Citrobacter rodentium* ICC180, carrying a luminescent tag, was streaked from −80°C stock on Luria agar containing 50 μg/ml kanamycin and 50 μg/ml nalidixic acid (LB + Kan + Nal) followed by overnight incubation at 37°C. A single colony was used to inoculate 25 ml of LB + Kan + Nal broth in a 50 ml tube in duplicate, followed by static overnight incubation at 37°C. Grown cultures were gently centrifuged (10 min at 5000 rpm), washed with PBS and resuspended in 2.5 ml of PBS.

Six animals were orally gavaged with 200 μl of *C. rodentium* lab grown cell suspension (see above) to increase infection by adapting bacteria to the GI environment. Stomach pH was not neutralized prior to infection. Weight and *C. rodentium* shedding by pre-infection animals was monitored on a daily basis. On day 9 post-gavage feces of infected animals were collected, re-suspended in PBS (1g in 10 ml), centrifuged (10 min at 4000 rpm), washed twice in PBS. Consequently, a 1 in 10 fecal solution containing *C. rodentium* cells (8.1 × 10^6^ CFU/ml) was obtained.

After 4 weeks of the diets, 48 animals were orally dosed with 200 μl of PBS (control animals) or 200 μl fecal solution containing 1.62 × 10^6^ CFU of *C. rodentium* (see above). Initially only 3 out of 24 animals were shedding *C. rodentium*, therefore at day six post-first dosing, animals received a second dose. In this case, PBS or 1.18 × 10^9^ CFU of *C. rodentium* cells grown in laboratory media (see above) were delivered via oral gavage. Following the second dose, all animals shed *C. rodentium.*

Murine feces were collected on a regular basis, followed by pellet resuspension in PBS in a 1:20 ratio. Re-suspended feces were serially diluted in PBS and 100 μl was duplicate plated on LB + Kan + Nal. This was followed by overnight incubation at 37°C and colony counting.

After 8 weeks of the diets, mice were sacrificed and trunk blood was collected. Organ lengths and weights were measured, followed by collection of cecum content for SCFA analysis. Distal fragments of colon and ileum of approximately 2.5 cm were Swiss rolled, dehydrated, paraffin embedded and stored until histology analysis. Two additional fragments of approximately 2.5 cm of colon and ileum were preserved in RNA later at −80°C for qPCR.

#### Histology

##### Tissue preparation for histology

Sections of colon and ileum (approximately 2.5 cm long) were rolled and kept overnight in 10% formalin, followed by 2 weeks in 70% ethanol for fixation. Tissue was then dehydrated and embedded in paraffin wax (100% ethanol 2.5 h; 100% ethanol 1.5 h; 1:1 100% ethanol:histolene 1 h; histolene 2 h; histolene 2 h; paraffin 2 h; paraffin 2 h) using Leica TP1020 Histokinette. Tissue was cut onto slides in 10 μm sections on a rotary microtome (Leica, RM2135), then stored at room temperature until histology commenced.

##### Measurement of colon crypt and ileum villi length

Sections of the colon and ileum were de-waxed using three 10 min incubations in Histochoice clearing agent (Amresco, H103). Sections were then rehydrated through a series of ethanol concentrations (100, 95, 90, 70%) for 2 min each, then rinsed with water for 5 min. Sections were then placed into Mayer’s Hematoxylin (VWR, 10047005) for 5 min, followed by a 5 min water rinse. Sections were then placed in Eosin (VWR, 10047003) for 30 s, rinsed with water, and dehydrated through a series of ethanol concentrations (70, 90, 95, 100%) for 2 min each. Sections were then placed in two 2 min incubations of Histochoice, then coverslipped with DPX mounting medium (Sigma, 06522). Sections were imaged at 40X using a brightfield microscope (Olympus DP71), and 5 crypt and 5 villi length were measured for each animal (*n* = 8 for healthy animals and *n* = 12 for *Citrobacter* infected animals) using ImageJ software (NIH), and the values were averaged per animal for each tissue type. All measurements were conducted in blind to groups.

#### Quantification of Fecal Lipocalin 2 (LCN2) by ELISA and Determination of Colitis Incidence

LCN2 levels were estimated in the fecal supernatants using Duoset murine LCN2 ELISA kit (R&D Systems, Minneapolis, Minnesota) as described previously (Chassaing et al., 2012), with following modifications. Frozen fecal samples were resuspended in PBS containing 0.1% Tween 20 to a final concentration of 100 mg/ml. A homogenous fecal suspension was generated by bead beating (FastPrep^®^-24, MP Biomedicals, United States) for a total of 20 min. These samples were then centrifuged (DragonLab, China) for 10 min at 14,000 × g and 4°C. Clear supernatants were collected and stored at −20°C until analysis. Ten to thousand time diluted samples were used in the assay according to the manufacturers’ instructions. The colorimetric peroxidase substrate tetramethylbenzidine was quantified by measurement of absorbance at 450 and 540 nm (SpectraMax M3, Molecular Devices). Analysis was done using four parameter logistic curve^1^.

#### Colon Cytokine Expression

##### RNA isolation from tissues

Approximately 30 mg of colon tissue preserved in RNAlater at −80°C was transferred into MagNA Lyser Green Beads (Roche) together with 600 μl of buffer RLT supplemented with 6 μl of 2-mercaptoethanol (β-ME). Tubes were subjected to MagNA Lyser (Roche) twice for 15 s at 6000. Lysate was centrifuged for 3 min at maximum speed (10,000 × *g*). Supernatant was mixed with 70% ethanol in 1 to 1 ratio. Sample, including any precipitate, was transferred onto RNeasy Mini spin column and centrifuged for 30 s at 10,000 × *g*. Column was washed with 700 μl of Buffer RW1 and twice with 500 μl Buffer RPE. RNA was eluted in 100 μl of RNase-free water (repeated elution). Isolated RNA sample was DNAse treated by adding 11 μl of 10X Turbo DNAse Buffer, 3 μl of Turbo DNAse (Ambion) and incubated for 30 min at 37°C. Second dose of 3 μl of Turbo DNAse was added followed by incubation for 30 min at 37°C. Next 22 μl of DNAse Inactivation Reagent was added and incubated for 5 min at room temperature to inactivate the enzyme. Finally, samples were centrifuged for 2 min at 10,000 × g, supernatant was transferred to fresh tubes and concentration of RNA was measured using Qubit BR RNA Assay Kit (Invitrogen/Thermo Fisher Scientific). RNA was stored at −80°C.

##### qPCR analysis of cytokine expression

Reverse transcription was performed in 10 replicates for each sample individually using 2 μg in 10 μl of RNA per reaction. Each reaction of 20 μl contained 4 μl of 5X Transcriptor buffer, 1 μl of 1:1 mix of Transcriptor Reverse Transcriptase (Roche) and Protector RNase Inhibitor (Roche), 2 μl of 10 mM dNTP mix (Roche), and 3 μl of Random Primers (Roche). Following the incubation for 10 min at 25°C, 30 min at 55°C, 5 min at 85°C and cooling to 4°C, replicates were pooled together for each of the samples. Resulting cDNA was used as a template in qPCR. Additionally, pooled RNA of all samples was processed without use of Transcriptase to serve as control for DNA contamination (No-RT sample).

Primers (Supplementary Table S1) were designed using Universal Probe Library Assay Design Center (Roche).

Each 10 μl reaction consisted of 5 μl of 2x LightCycler 480 Probes Master (Roche), 0.5 μl of each of 10 mM primers, 0.1 μl of Universal ProbeLibrary (UPL) Probe (Roche) and 2 μl of template. No template controls (NTC) were prepared using water as template. Each reaction was run in quadruplicates. Reactions were run on a 384-well LightCycler 480 PCR plates and sealed using LightCycler 480 adhesive cover both supplied by Roche. Cycling parameters were 95°C for 10 min, followed by 55 cycles of 95°C for 10 s and 60°C for 45 s. Signal intensity was measured at 72°C for 1 s. Crossing point (Cp) values were calculated automatically using instrument software.

All target genes were considered as potential normalization genes and were evaluated according to the criteria defined by GeNorm (Vandesompele et al., 2002). Expression was normalized using geometric means of 2 (V_2__/__3_ = 0.128) highly stable genes GAPDH (*M* = 0.364) and Tbp (*M* = 0.348). The 2^–ΔΔ*CT*^ method (Livak and Schmittgen, 2001) was used to normalize the data. Normalized values were represented in relation to expression in PBS-dosed animals on standard or ADR-159 diet. Statistical analysis, one-way ANOVA with correction for multiple testing, was performed using qbase + software (Hellemans et al., 2007).

#### SCFA Analysis

Fecal water was prepared by homogenizing the cecum content (~150–200 mg) with 1 ml of acidic MilliQ water (10,000 time diluted 36.5–38% hydrochloric acid in Milli-Q water) using a vortex mixer for 5 min. The homogenized samples were centrifuged at 15,000 rpm for 5 min and the supernatants were filtered with 0.22 μM filters (Corning). Duplicate aliquots of 270 μl of filtrate were mixed with 30 μl of 10 mM 2-ethyl-butyric acid that was used as internal standard. The samples were homogenized briefly followed by a further centrifugation step of 15,000 rpm for 3 min. Finally, the supernatant was transferred to 250 μl inserts (Agilent) placed in amber glass 2 ml GC vials (Agilent) sealed with silicone/PTFE screw caps (Agilent).

A Varian 3800GV system, fitted with an Agilent DB-FFAP column (30 ml × 0.32 mm ID × 0.25 μm df) and a flame ionization detector with a CP-8400 auto-sampler was used to analyze standards and samples by gas chromatography flame ionization detection (GC-FID). A flow rate of 1.3 ml/min was used with helium as a carrier gas. An initial oven temperature of 50°C was maintained for 30 s, then raised to 140°C at 10°C/min and held again for 30 s. Finally, the temperature was increased to 240°C at 20°C/min, and held for 5 min (for a total run time of 20 min). The temperatures of the detector and the injection port were 300 and 240°C, respectively. A 10 μl syringe (Agilent) installed to a CP-8400 auto-sampler (Varian) was used to perform a split-less injection of 0.2 μl for each sample or standard. Peak integration was performed with Varian Star Chromatography Workstation version 6.0 software. To check for any potential carryover vials containing 1800 μl of water were run between each sample duplicates as blanks. Standards were included in each run to maintain the calibration.

A 7 point standard curve was generated (SCFA 0.1, 0.5, 1, 2, 4, 8 and 10 mM; BCFA 0.01, 0.05, 0.1, 0.2, 0.4, 0.8 and 1 mM) in acidic Milli-Q water with 1 mM internal standard. Results are expressed as mean of two technical replicates (except samples B1, B4, B6, C3, C5, D6, D10, D11) calculated to μM per gram of wet mass. Samples A7 and C7 had a mass too low to process.

#### Statistical Analysis for Physiological Responses

Statistical analyses were conducted using SPSS software (IBM Corporation). Data that were normally distributed according to Shapiro–Wilk test or that had homogeneous variances were analyzed using parametric tests, one-way ANOVA or repeated measures ANOVA. When required a *post hoc* analysis was performed using Bonferroni or Games-Howell correction. Not normally distributed data violating the condition of homogeneity of variances were analyzed using the non-parametric Kruskal–Wallis test. Statistical significance was set at *p* < 0.05.

### 16S rRNA Gene Metagenomics

#### DNA Isolation, Amplification, Indexing, Normalization, and Sequencing

DNA isolation and library preparation were conducted as previously described (Warda et al., 2018). The QIAamp Fast DNA Stool mini kit (Qiagen) was used according to the manufacturers’ recommendations to isolate DNA from fecal samples stored until processing at −80°C. DNA concentration was measured using a Qubit dsDNA HS Assay Kit and DNA quality was assessed by running a 5 μl sample on a gel. Phusion Polymerase Master Mix and V3-V4 (Forward 5′-TCGTCGGCAGCGTCAGATGTGTATAAGAGACAGCCTA CGGGNGGCWGCAG-3′; Reverse 5′-GTCTCGTGGGCTCG GAGATGTGTATAAGAGACAGGACTACHVGGGTATCTAAT CC-3′) primers were used to amplify the V3 and V4 region of 16S rRNA genes (98°C 30 s; 25 cycles of 98°C 10 s, 55°C 15 s, 72°C 20 s; 72°C 5 min). Quality and quantity of the amplicons were checked by Qubit dsDNA HS Assay Kit and by running on a gel. This was followed by amplicon cleanup using Ampure XP magnetic beads. Five microliter of cleaned amplicon was used as a template for Index PCR using Phusion Polymerase Master Mix and Nextera XT Index Kit (95°C 30 s; 8 cycles of 95°C 30 s, 55°C 30 s, 72°C 30 s; 72°C 5 min). The resulting Indexed amplicons were cleaned using Ampure XP magnetic beads and their quality and quantity were checked by Qubit dsDNA HS Assay Kit and running on gel, respectively. Finally, all samples were normalized to 4 nM, followed by pooling 5 μl of each sample for Illumina MiSeq sequencing by GTAC (Germany).

#### 16S rRNA Gene Data Analysis

First FastQC was used to examine the quality of raw sequences followed by filtering and trimming using Trimmomatic (Bolger et al., 2014) to ensure only high quality reads were retained for further analysis. Briefly, both forward and reverse reads were subjected to the removal of the first 15 bases while the forward was cropped at 240 bases and the reverse at 225. Next, we filtered reads using a sliding window (4:20) with the minimum read length set to 200 bases. Subsequently, the DADA2 pipeline (Callahan et al., 2016) was used to process and merge the remaining high quality reads. ChimeraSlayer and the Gold database (Edgar et al., 2011) was used for further chimera filtering of the resulting Ribosomal Sequence Variants (RSVs). Sequences were classified from phylum to genus using the Mothur implementation of the Ribosomal Database Project (RDP) classifier and the RDP database (v11.4) with assignments obtaining a bootstrap value of less than 80% assigned as the preceding rank and unclassified. SPINGO against the RDP (v11.4) with default parameters (similarity score of 0.05 and a bootstrap cut-off of 0.8) (Allard et al., 2015) was used for species classification.

All downstream analysis (and microbiome figures) were generated in R (v3.5.1). The phyloseq package (McMurdie and Holmes, 2013) was used to calculate alpha and beta diversity. Alpha diversity comparisons were performed using a Mann-Whitney test, while beta diversity was via an Adonis test from the vegan package. The differential abundance of the genera and RSVs was carried out with DESeq2 (Love et al., 2014). Analysis was performed separately for the initial effect of the diet (weeks 0, 2, 3, and 4) and the effect of the dosing (before dosing/week 4, 8, 12, and 18 days post dosing).

### Virome Analysis

#### Fecal Virus-Like Particles (VLPs) Nucleic Acid Extraction

Pooled fecal samples of 0.2–1.5 g per cage were stored at -80°C until processing according to a previously published method (Shkoporov et al., 2018). In brief, feces were resuspended in 10 ml of SM buffer and vortexed for 5 min. Following 5 min incubation on ice tubes were centrifuged at 5000 rpm in a swing bucket rotor for 10 min at 4°C. Supernatants were transferred to new tubes, and centrifugation was repeated once again. A 0.45 μm pore PES syringe-mounted membrane filters were used to filter supernatant; this was repeated once. The filtrates were first supplemented with 5M NaCl solution to a final concentration of 0.5 M, next Polyethylene glycol (PEG-8000) powder was added to a final concentration of 10% w/v. Following the resuspension, samples were kept for 16 h at 4°C.

The precipitate was collected by centrifugation of samples at 5000 rpm for 20 min at 4°C. The supernatant was discarded, and any residual supernatant was removed by 5 min incubation of the tubes in an inverted position on paper towels. Pellets were then resuspended in 400 μl of SM buffer, supplemented with 400 μl of chloroform and extracted by gentle shaking. A desktop centrifuge (2500 g for 5 min) was used to facilitate phase separation. The aqueous phase (∼360 μl) was transferred into fresh tubes and mixed with 40 μl of a solution of 10 mM CaCl_2_ and 50 mM MgCl_2_. DNA/RNA digestion was carried out at 37°C for 1 h by addition of 16 U of TURBO DNase (Ambion/Thermo Fisher Scientific) and 20 U of RNase I (Thermo Fisher Scientific). Digestion enzymes were heat inactivated at 70°C for 10 min. 2 μl of Proteinase K (Qiagen) and 20 μl of 10% SDS were then added to the tubes, and incubation was continued for 20 min at 56°C. 100 μl of Phage Lysis Buffer (4.5 M guanidinium isothiocyanate, 44 mM sodium citrate pH 7.0, 0.88% sarkosyl, 0.72% 2-mercaptoethanol) was added and kept at 65°C for 10 min to lyse the viral particles. Finally, lysates were extracted by gentle vortexing with equal volume of Phenol/Chloroform/Isoamyl Alcohol 25:24:1 (Fisher Scientific) followed by centrifugation at 8,000 × g for 5 min at room temperature. The phenol/chloroform extraction step was repeated. DNeasy Blood & Tissue Kit (Qiagen) was used to purify the resulting aqueous phase according to manufacturer’s instruction, with a final elution volume of 50 μl.

As a negative control, the procedure was performed with no fecal samples.

#### Shotgun Sequencing of Fecal VLP Nucleic Acids

Reverse transcription, multiple displacement amplification (MDA) and library preparation were conducted according to a previously published method (Shkoporov et al., 2018). SuperScript IV Reverse Transcriptase (RT) kit (Invitrogen/ThermoFisher Scientific) was used according to the manufacturer’s random hexamer primer protocol. More specifically eleven microliters of fecal VLP nucleic acid sample regardless of concentration was used for the reverse transcription reaction. One microliter of reverse-transcribed nucleic acids was then amplified using MDA technology with Illustra GenomiPhi V2 kit (GE Healthcare). The latter step was done in triplicate for each sample. The remainder of RT products (17 μl) and the products from all three MDA reactions were pooled together and subjected to additional round of purification using DNeasy Blood & Tissue Kit.

Qubit dsDNA HS Assay Kit (Invitrogen/Thermo Fisher Scientific) was used to quantify the amplified DNA. Nextera XT Nano DNA Library Preparation Kit (Illumina) was used for the random shotgun library preparation and bead-based normalization following the standard manufacturer’s protocol. Ready-to-load libraries were sequenced using a proprietary modified protocol using 2 × 300 bp paired-end chemistry on an Illumina MiSeq platform (Illumina, San Diego, CA, United States) at GATC Biotech AG, Germany.

#### Virome Data Analysis

Raw sequence quality was accessed using FASTQC while reads were filtered using the following parameters; HEADCROP:10, CROP: 125 and 120 bp for forward and reverse respectively; SLIDINGWINDOW: 4:20 and a MINLEN of 50 bp. Read assembly of the retained high quality reads was carried out per sample using SPAdes in metagenomic mode (Nurk et al., 2017). Assemblies were pooled and all contigs greater than 1 kb were retained. Redundancy was removed with 90% identity over 90% of the length (of the shorter) retaining the longest contig in each case. Using a set of criteria, bacterial contamination was removed thereby retaining only viral genomes or partial genomes. Briefly, a contig must meet at least one of the following; (1) alignment to an in-house crAssphage database (Guerin et al., 2018) (threshold: 1e-10), (2) a minimum of 2 pVogs with at least 3 per 1 kb (Grazziotin et al., 2017), (3) VirSorter positive (Roux et al., 2015), (4) circular, (5) greater than 3 kb with no hits to the NT database (January 19) (threshold: 1e-10), (6) hits to viral RefSeq database (v.89) (threshold: 1e-10), and finally, (7) less than 3 ribosomal proteins as predicted using the COG database (Tatusov et al., 2000). Post-filtering, high quality reads were aligned to the reference set of viral contigs (*n* = 5,638) using bowtie2. SAMtools was employed to generate a count table and a 75% breadth of coverage filter was utilized to prevent false positives from spurious alignments to repeat regions. Any counts which did not meet the 75% threshold were set to 0. This resulted in a final set of 5,016 viral contigs.

Alpha and beta diversity analysis were performed separately for the initial effect of the diet (weeks 0, 2, and 4) and the effect of the dosing (before dosing/week 4, 8, 12, and 18 days post-dosing).

## Results

### ADR-159 Diet Subtly Modifies the Murine Microbiome

**FIGURE 2:**
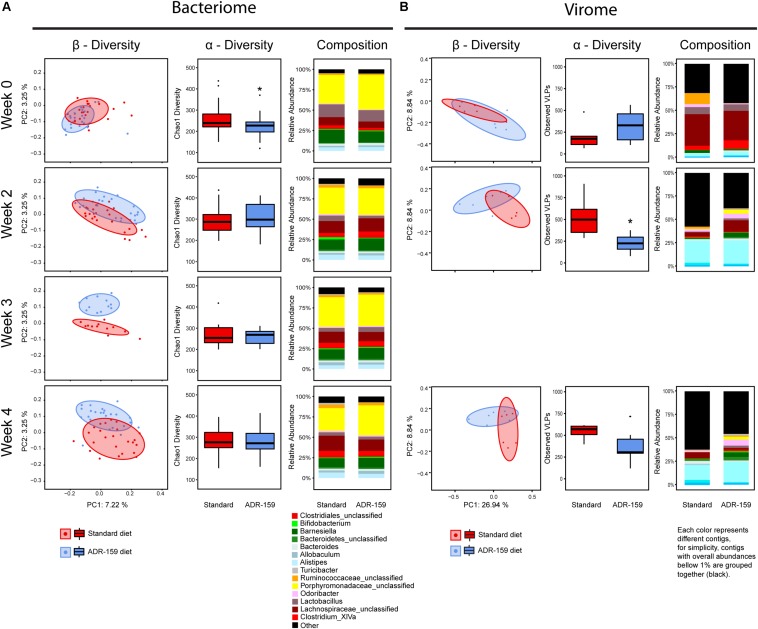
Microbiome analysis before (week 0) and during (weeks 2, 3, and 4) the diet intervention with standard (red) and ADR-159 (blue) diet. **(A)** Bacteriome. 24 animals are represented per group at weeks 0, 2, and 4, while only 12 animals per group in week 3. Left: PCoA plots of microbiome composition using Jaccard distances. Each dot represents individual animal at each given time point. Ellipses represent the confidence interval of every group at 75%. Middle: Chao1 index, * indicates significant difference. Right: mean abundance at genus level. For simplicity, genera with abundances below 1% were grouped together. **(B)** Virome. Each group represents 6 cages, each of 4 animals. Left: PCoA plots of virome composition using the Spearman distances. Each dot represents a cage with 4 individual animals at each given time point. Ellipses represent the confidence interval of every group at 75%. Middle: observed number of viral contigs, * indicates significant difference. Right: mean abundance of viral contigs. Each color represents different contig, for simplicity, contigs with overall abundances below 1% were grouped together (black).

Bacteroidetes and Firmicutes were the dominant phyla in the microbiome of both standard and ADR-159 fed animals (data not shown), with no clear difference between groups throughout the experiment. At the genus level, the microbiome composition of both cohorts was more diverse, with a high contribution of unclassified *Porphyromonadaceae* (approximately 45% in each of the samples) (Figure 2A and Supplementary Figure S2). This absence of radical differences in microbiome composition between the two groups of animals indicates that extended consumption of ADR-159 did not result in a preeminent disruption of the microbiome of healthy animals, but did cause subtle statistically significant modifications over the initial 4-week period (Figure 2A) (ADONIS; *p* = 0.002, *p* = 0.001, *p* = 0.002, *p* = 0.001). Still, over 10% of variability shown on Figure 2A could be explained by combination of PC1 and PC2 axes. More specifically, in ADR-159 fed animals we observed a stable reduction of *Turicibacter*, *Clostridium sensu strict*, and *Dorea* (Table 1). To investigate subtle changes in microbiome composition we also performed analysis at the Ribosomal Sequence Variants (RSV) level. Only 22 RSVs were differentially abundant, supporting the initial observations. Four of those RSVs could be assigned to species level (*Bifidobacterium pseudolongum*, *Clostridium disporicum*, *Clostridium ruminantium*, *Turicibacter sanguinis*, Supplementary Table S2).

**TABLE 1:** Genera differently abundant during 4 weeks of ADR-159 feeding compared to animals fed a standard diet.

**Genus**	**Week 0**			**Week 2**			**Week 3**			**Week 4**			**Effect in ADR-159 diet group**
				
	**Mean control diet (AB)**	**Mean ADR-159 diet (CD)**	***p*-value adjusted**	**Mean control diet (AB)**	**Mean ADR-159 diet (CD)**	***p*-value adjusted**	**Mean control diet (AB)**	**Mean ADR-159 diet (CD)**	***p*-value adjusted**	**Mean control diet (AB)**	**Mean ADR-159 diet (CD)**	***p*-value adjusted**	
*Bifidobacterium*				2.77E-02	7.54E-03	4.71E-03							Temporary ↓
*Clostridiales* unclassified				1.87E-02	3.29E-02	4.01E-02							Temporary ↑
*Clostridium* sensu stricto				3.35E-03	2.51E-04	1.46E-05	4.33E-03	1.85E-05	1.30E-02	6.03E-03	5.54E-04	5.04E-06	↓
*Clostridium* XI				1.53E-03	3.20E-05	4.95E-02							Temporary ↓
*Clostridium* XlVb										2.16E-03	9.13E-04	6.69E-03	Temporary ↓
*Desulfohalobiaceae* unclassified							7.20E-03	1.89E-03	3.12E-02				Temporary ↓
*Dorea*				1.73E-03	7.19E-04	2.56E-02				2.69E-03	1.17E-03	1.01E-02	↓
*Enterobacteriaceae* unclassified				1.03E-04	6.59E-04	4.95E-02							Temporary ↑
*Eubacterium*										9.03E-05	2.97E-04	4.00E-02	Temporary ↑
*Lachnospira*										1.13E-03	2.32E-04	3.37E-04	Temporary ↓
*Lactobacillus*				6.79E-02	3.16E-02	2.56E-02							Temporary ↓
*Mucispirillum*	2.57E-03	3.18E-04	9.63E-03										Recovery from initially lower level
*Porphyromonadaceae* unclassified										2.77E-01	3.66E-01	6.69E-03	Temporary ↑
*Prevotellaceae* unclassified										4.99E-03	8.09E-03	1.01E-02	Temporary ↑
*Turicibacter*				1.13E-02	2.66E-03	4.29E-04				2.08E-02	5.04E-03	5.04E-06	↓

The compositional analysis described above (β-diversity) illustrates the degree of difference in the microbial composition of animals on the different diets. The α-diversity, which is presented below, describes the number of different species detected in animals on a given diet.

The α-diversity of the microbiome, evaluated using both Chao1 (Figure 2A) and Shannon indexes (data not shown), did not undergo consistent changes during the trial. Before diet differentiation, animals assigned to the ADR-159 group had a slightly reduced α-diversity compared to the standard diet group, nevertheless this difference became insignificant by week 2 of the trial. Similarly, the microbiome of animals at week 0 differed slightly between animals randomly assigned to standard and ADR-159 fed groups. Initially (at week 0) animals in the ADR-159 group had lower abundance of *Mucispirillum* compared to the standard-fed group (Table 1), this difference was not observed over the following weeks, suggesting that the initial differences were due to chance. Other studies have also reported initial differences in limited numbers of taxa/OTUs between groups (Holder et al., 2019). Although we focus on the effect of the diet at the given time point, it is important to note that the microbiome of animals on both diets exhibited compositional changes and changes in β-diversity during the course of the experiment (Supplementary Figure S2) indicating the dynamic nature of the microbiome.

#### Virome Analysis Confirms Subtle Nature of Microbiome Changes in ADR-159 Fed Animals

Virome analysis was performed per cage rather than per individual. Prior to diet diversification, virome β-diversity was comparable between the standard and ADR-159 fed animals (ADONIS, *p* = 0.4645). Over time, the separation between the viromes of the two groups reached significance at week 4 (ADONIS, *p* = 0.04795). A considerably higher percentage of variability was explained by virome PC1 and PC2, 26.94 and 8.84%, respectively (Figure 2B) compared to 16S. Virome α-diversity in standard fed animals increased after diet diversification (*p* = 0.0185), while in ADR-159 fed animals it remained stable with approximately 250 observed viral contigs. After 2 weeks virome α-diversity was significantly higher in standard fed animals compared to those receiving ADR-159 (*p* = 0.01515) (Figure 2 and Supplementary Table S4).

The viral contig composition dramatically changed during the initial 2 weeks independent of the diet, indicating that despite acclimatization animals on both standard and ADR-159 diets were still undergoing change, probably at a level under the discriminatory power of 16S rDNA analysis (Figure 2B). As many as 56 viral contigs were differently abundant in at least two time points, while 94, 279, and 127 viral contigs were differentially abundant at single time point (weeks 0, 2, and 4, respectively; data not shown). A higher compositional diversity was observed between virome samples (Supplementary Figure S3) compared to individual 16S samples.

Nevertheless, the virome of animals in both groups underwent similar changes, corroborating a lack of major disruption of the microbiome in the ADR-159 fed animals and supporting a more subtle change in less abundant taxa and at sub-OTU level.

### Effect of ADR-159 Diet Modification of Microbiome on *Citrobacter* Infection

**FIGURE 3:**
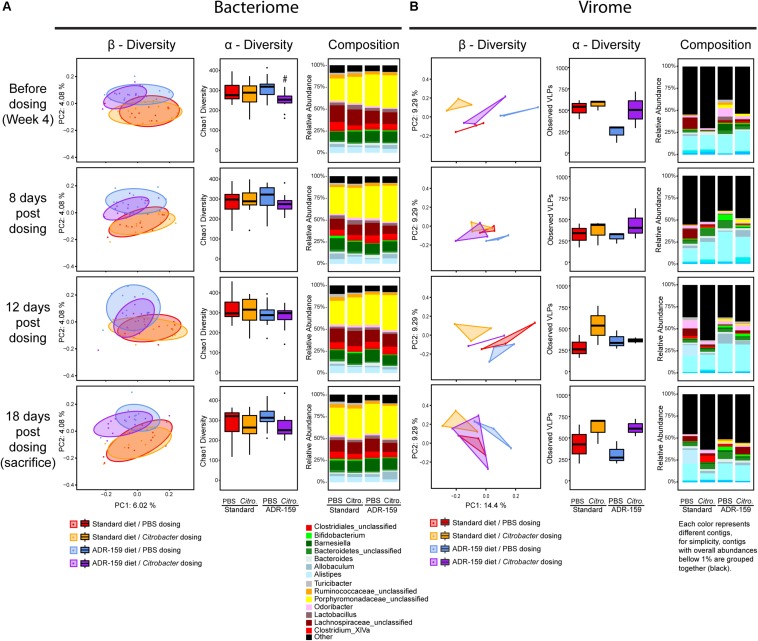
Microbiome analysis of standard (red and orange) and ADR-159 (blue and purple) fed animals before and 8, 12, and 18 days post-initial dosing with PBS (red and blue) and *Citrobacter* (orange and purple). **(A)** Bacteriome. Each group represents 12 animals. Left: PCoA plots of microbiome composition using Jaccard distances. Each dot represents individual animal at each given time point. Ellipses represent the confidence interval of every group at 75%. Middle: Chao1 index. Right: mean abundance at genus level. For simplicity, genera with abundances below 1% were grouped together. **(B)** Virome. Each group represents 3 cages, each of 4 animals. Left: PCoA plots of virome composition using Spearman distances. Each dot represents cage of 4 individual animal at each given time point. For visualization, a triangle area was imposed representing the presence of up to 3 samples per group. Middle: observed number of viral contigs, * indicates significant difference. Right: mean abundance of viral contigs. Each color represents different contig, for simplicity, contigs with overall abundances below 1% were grouped together (black).

At week 4, half of the animals on each diet were orally dosed with *Citrobacter* while the other half were dosed with PBS to serve as a control. All mice continued on the same diets for the remainder of the experiment. Despite the initiation of an intensive animal handling phase of the trial, the microbiome of ADR-159 fed animals maintained the reduction of *Turicibacter* and *Clostridium sensu stricto* as well as an increase of unclassified *Prevotellaceae* that began at week 4 (Table 2). Additional stable changes (two consecutive time points) included a reduction in unclassified *Bacteroidetes* and *Clostridium* XI in ADR-159 fed animals (Table 2). These changes may reflect not only response to extended exposure to the diet but rather a combination of diet and animal handling. Still, over 10% of bacteriome variability following dosing could be explained by the combination of PC1 and PC2 axes (Figure 3A).

**TABLE 2:** Genera differently abundant before and 8, 12, and 18 days post-initial dosing with PBS (A, C) and *Citrobacter* (B, D) of standard (A, B), and ADR-159 (C, D) fed animals.

**Difference**	**Condition 1**	**Condition 2**	**Genus**	**Before dosing (week 4)**			**8 days post-dosing**			**12 days post-dosing**			**18 days post-dosing (sacrifice)**			**Effect**
							
				**Mean 1**	**Mean 2**	**p-value adjusted**	**Mean 1**	**Mean 2**	**p-value adjusted**	**Mean 1**	**Mean 2**	**p-value adjusted**	**Mean 1**	**Mean 2**	**p-value adjusted**	
Difference in diet - no infection	Standard diet/PBS dosing	ADR-159 diet/PBS dosing	*Allobaculum*				6.37E-02	1.08E-02	2.79E-03							Temporary ↓ in ADR-159
			*Anaeroplasma*				6.29E-04	3.96E-05	3.32E-02							
			*Bacteroidales* unclassified							1.77E-03	3.18E-03	1.21E-02				Temporary ↑ in ADR-159
			*Bacteroidetes* unclassified							2.93E-02	1.45E-02	1.82E-03	2.84E-02	1.81E-02	1.10E-02	↓ in ADR-159
			Bifidobacterium*				2.83E-02	4.00E-03	1.44E-03							
			Candidatus *Saccharibacteria* unclassified							1.80E-03	3.97E-04	1.95E-03				Temporary ↓ in ADR-159
			*Clostridia* unclassified							7.34E-04	1.50E-04	4.79E-02				
			Clostridium sensu stricto*	6.96E-03	3.65E-04	1.06E-02	5.14E-03	7.34E-04	1.13E-02	6.43E-03	3.34E-04	1.95E-03	5.32E-03	8.03E-04	8.07E-03	↓ in ADR-159
			*Clostridium* XI*				1.37E-03	0.00E+00	3.31E-02	1.21E-03	0.00E+00	2.90E-03	1.14E-03	1.92E-04	1.10E-02	
			*Deltaproteobacteria* unclassified							5.77E-04	1.33E-04	3.43E-02				Temporary ↓ in ADR-159
			*Flavonifractor*							1.41E-02	5.05E-03	1.82E-03				
			*Porphyromonadaceae* unclassified*	2.53E-01	3.62E-01	4.40E-02										↑ in ADR-159
			*Prevotellaceae* unclassified*							6.16E-03	1.46E-02	1.76E-03				
			*Ruminococcus*										1.38E-02	5.90E-03	2.03E-02	Temporary ↓ in ADR-159
			Turicibacter*	2.00E-02	5.02E-03	1.06E-02	2.18E-02	3.22E-03	1.44E-03	3.46E-02	6.35E-03	2.42E-03	2.89E-02	7.28E-03	8.65E-03	↓ in ADR-159
Same diet (ADR-159) - difference in infection	ADR-159 diet/PBS dosing	ADR-159 diet/ *Citrobacter* dosing	*Alistipes*										6.06E-02	3.22E-02	8.51E-03	Temporary ↓ in *Citrobacter*
			*Allobaculum*				1.08E-02	4.98E-02	2.82E-03							Temporary ↑ in *Citrobacter*
			*Bacteroides*				3.72E-02	1.65E-02	1.41E-02	3.89E-02	1.21E-02	1.41E-03				↓ in *Citrobacter*
			*Prevotellaceae* unclassified							1.46E-02	5.89E-03	1.41E-03				Temporary ↓ in *Citrobacter*
Same diet (standard) - difference in infection	Standard diet/PBS dosing	Standard diet/ *Citrobacter* dosing	*Anaeroplasma*							1.02E-03	1.02E-05	2.93E-02				Temporary ↓ in *Citrobacter*
			*Bacteroides*							2.68E-02	1.00E-02	2.90E-04				
			*Bacteroidetes* unclassified							2.93E-02	1.81E-02	1.20E-02				
			*Citrobacter*							0.00E+00	6.32E-03	1.21E-02				Temporary ↑ in *Citrobacter*
			*Flavonifractor*							1.41E-02	6.85E-03	1.20E-02				Temporary ↓ in *Citrobacter*
			*Vampirovibrio*							2.71E-04	1.51E-03	6.68E-03				Temporary ↑ in *Citrobacter*
Difference in diet - all infected	Standard diet/ *Citrobacter* dosing	ADR-159 diet/ *Citrobacter* dosing	*Akkermansia*										4.88E-03	2.84E-02	4.87E-02	Temporary ↑ in ADR-159
			*Alistipes*	6.74E-02	4.46E-02	2.64E-02				6.88E-02	4.11E-02	3.36E-02	5.92E-02	3.22E-02	1.56E-02	↓ in ADR-159
			*Bacteroidales* unclassified							1.40E-03	3.92E-03	2.15E-02				Temporary ↑ in ADR-159
			*Clostridium* sensu stricto*	5.10E-03	7.43E-04	7.82E-03	4.70E-03	7.59E-04	5.63E-03	4.04E-03	6.88E-04	2.15E-02	7.90E-03	4.89E-04	5.90E-03	↓ in ADR-159
			*Clostridium* XI*										1.51E-03	0.00E+00	2.10E-03	Temporary ↓ in ADR-159
			*Clostridium* XlVb*	1.97E-03	4.86E-04	2.52E-02										
			Dorea*										2.80E-03	8.56E-04	4.87E-02	
			*Flavonifractor*										8.95E-03	4.42E-03	4.87E-02	
			Lachnospira*	1.00E-03	1.83E-04	2.52E-02										
			*Proteobacteria* unclassified										4.08E-03	1.33E-03	4.73E-02	
			*Ruminococcaceae* unclassified										4.10E-02	2.18E-02	4.07E-02	
			Turicibacter*	2.16E-02	5.06E-03	7.82E-03	1.67E-02	6.50E-03	5.63E-03				2.29E-02	6.85E-03	1.56E-02	↓ in ADR-159
			*Vampirovibrio*							1.51E-03	2.99E-04	2.15E-02				Temporary ↓ in ADR-159

*Indicated taxa significantly different at one of the initial 4 weeks of experiment.

Overall, prior to dosing animals with *Citrobacter* or PBS, the microbiome of the animals differed (β-diversity and composition) as a result of the 4-week periods of standard or ADR-159 diet (Figure 2). Throughout the infection process (8-, 12-, and 18-days post-initial dosing) this clear division between the microbiomes of standard and ADR-159 fed animals based on β-diversity and composition was maintained (Figure 3A). *Citrobacter* infection did not cause a major disruption to the microbiome (Figure 3A and Supplementary Figure S2), but *Citrobacter* was detected in the microbiome of a majority of *Citrobacter* dosed animals at levels of approximately 0.2%. In animals on both diets *Citrobacter* infection coincided with a reduction in *Bacteroides*, while the impact of dosing on other genera depended on the diet (Table 2 and Supplementary Table S3). *Citrobacter* infected animals on standard diet had increased levels of *Citrobacter* and *Vampirovibrio*, and reduced levels of *Anaeroplasma*, unclassified *Bacteroidetes* and *Flavonifactor* (Table 2 and Supplementary Table S3) as compared to healthy controls. At the same time, *Citrobacter* infected animals on the ADR-159 diet had increased levels of *Allobaculum* and reduced levels of *Alistipes* and unclassified *Prevotellaceae*, as compared to uninfected controls.

There was no major effect of *Citrobacter* dosing on α-diversity as evaluated by both Chao1 (Figure 3A) and Shannon indexes. Before infection animals assigned to the ADR-159/*Citrobacter* group had reduced Chao1 diversity compared to ADR-159/PBS group but this difference was not maintained post-dosing.

#### Impact of ADR-159 on Virome of Mice Following *Citrobacter* Infection

Following dosing with PBS or *C*. *rodentium*, virome analysis was continued per cage, with 3 cages per group (Figure 3B). Following the dosing, numbers of observed viral contigs for healthy standard fed animals continued to fluctuate, as it did during the initial 4 weeks of the experiment (Figure 3B). At the same time, numbers of observed viral contigs for PBS-dosed ADR-159 fed animals remained stable. Overall, higher numbers of viral contigs were observed in animals dosed with *Citrobacter* compared to healthy controls, however, statistical significance was limited by low sample numbers as virome analysis was done per cage, resulting in only three samples per group (Supplementary Table S5).

Virome composition revealed higher diversity when compared to 16S data, which may be attributed to the pooled nature of the collected samples and the fact that low abundance contigs (less than 1%) represent approximately 50% of the total (Figure 3B). None of the viral contigs could be associated with *Citrobacter* infecting phages or *Citrobacter* hosts.

### ADR-159 Diet Has No Effect on Body Weight

**FIGURE 4:**
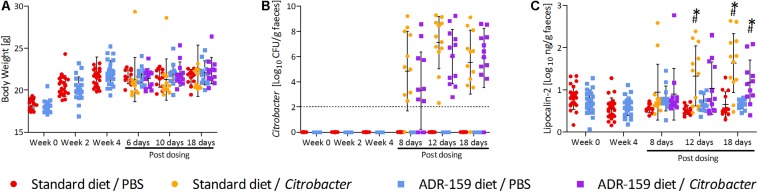
Measurements for standard (circle) and ADR-159 (square) fed animals dosed with *C*. *rodentium* (orange, purple) or PBS (red, blue) before start of the diet, after 4 weeks of the diet (before dosing) and up to 18 days post-dosing (sacrifice). Error bars represent SD. **(A)** Body weight. **(B)** Average *C*. *rodentium* levels shed before and after initial dosing. **(C)** Fecal lipocalin-2 (LCN2) levels. ^∗^ Indicates significant change compared to standard diet/PBS, # indicates significant change compared to ADR-159/PBS.

There was no difference in initial body weight of animals assigned to receive standard or ADR-159 diet (Mann–Whitney *U*-test, *p* = 0.317). While the body weight of all animals changed over time [repeated measures ANOVA, Greenhouse-Geisser; *F*(3, 253) = 137.887, *p* < 0.0005], there was no difference in body weight of standard and ADR-159 fed animals dosed with either PBS or *Citrobacter* [repeated measures ANOVA, Greenhouse–Geisser; *F*(9, 758) = 1.547, *p* = 0.131; Figure 4]. Therefore, it was stipulated that a sufficient caloric intake was provided with *ad libitum* ADR-159 diet.

### ADR-159 Diet Does Not Prevent *Citrobacter* Infection

After 4 weeks animals on both diets were orally dosed with either *Citrobacter* or PBS. Comparable levels of *Citrobacter* shedding were observed for both standard and ADR-159 fed animals [Greenhouse–Geisser test, time^∗^treatment, *F*_(__11__)_ = 1.1418, *p* = 0.332; Figure 4]. A high heterogeneity was observed in both standard and ADR-159 fed animals regarding the initiation and rate of infection. However, in both groups the proportion of infected animals (as defined by shedding 10^6^ cfu/g feces) was not significantly different [χ^2^(1) = 0.9709, *p* = 0.325) (Supplementary Figure S1). No *Citrobacter* was detected in the feces of PBS-dosed animals or animals before dosing.

### Impact of ADR-159 and *Citrobacter* Infection on Inflammation

The ADR-159 diet had no effect on fecal lipocalin 2 (LCN2) levels over the initial 4 weeks of the trial (independent *t*-test; week 0 *t* = 1.354, *p* = 0.183; week 4 *t* = -0.852, *p* = 0.399). Following the *Citrobacter* dosing significant changes were detected between groups after 12 [ANOVA, *F*(3, 44) = 6.686, *p* = 0.001] and 18 days [ANOVA, *F*(3, 43) = 12.203, *p* < 0.0005]. At 12 days post-dosing animals on standard diet dosed with *Citrobacter* had significantly increased LCN2 levels (*post hoc* Games-Howell, *p* = 0.011). At the same time there was no difference between *Citrobacter* and PBS dosed animals on the ADR-159 diet (*post hoc* Games-Howell, *p* = 0.272), nor between standard and ADR-159 fed animals dosed with PBS (*post hoc* Games-Howell, *p* = 0.113). At sacrifice both standard (*post hoc* Games–Howell, *p* = 0.002) and ADR-159 (*post hoc* Games–Howell, *p* = 0.040) fed animals dosed with *Citrobacter* had significantly higher LCN2 levels compared to their PBS-dosed counterparts (Figure 4).

#### ADR-159 Prevents a Reduction of Small Intestine Length in *Citrobacter* Infected Animals

**FIGURE 5:**
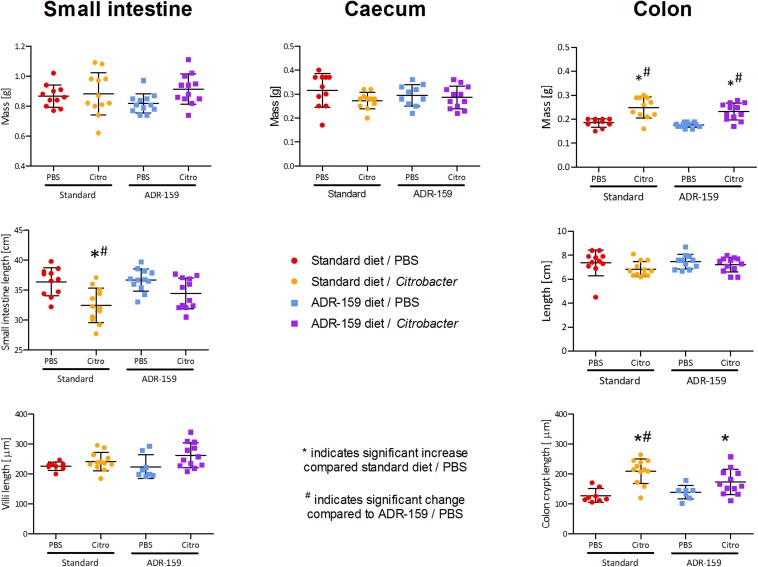
Mass, total length, and villi/crypt length of internal organs of standard (circle) and ADR-159 (square) fed animals dosed with PBS (red, blue) or *C*. *rodentium* (orange, purple). Error bars represent SD. *Indicates significant increase compared to standard diet/PBS, ^#^Indicates significant change compared to ADR-159/PBS.

The length of the small intestine differed between groups [*F*(3, 43) = 7.681, *p* < 0.0005; Figure 5]. *Citrobacter* infection led to a reduction of small intestine length in standard fed animals (*post hoc* Bonferroni, *p* = 0.002). In ADR-159 fed animals this reduction was not observed (*post hoc* Bonferroni, *p* = 0.188). There was no significant difference in the mass of the small intestine [*F*(3, 43) = 1.880, *p* = 0.147], cecum [*F*(3, 40) = 1.354, *p* = 0.271] or colon length [*F*(3, 42) = 1.525, *p* = 0.222]. In both standard and ADR-159 fed animals the colon weight increased in *Citrobacter* infected animals compared to healthy controls (Kruskal–Wallis Test, *p* < 0.0005; *post hoc* Mann–Whitney *U*-test, *p* < 0.0005 for both diets).

#### ADR-159 Prevents an Increase of Colon Crypt Length in *Citrobacter* Infected Animals

Colon crypt length differed between groups [*F*(3, 36) = 10.552, *p* < 0.0005; Figure 5]. In standard fed animals the length of colon crypts increased in the *Citrobacter* infected animals (*post hoc* Bonferroni, *p* < 0.0005). In ADR-159 fed animals the colon crypt length was not affected by *Citrobacter* infection (*post hoc* Bonferroni, *p* = 0.267). At the same time the length of ileum villi was not affected by infection and/or diet (Kruskal–Wallis Test, *p* = 0.052).

#### Colon Cytokine Expression Levels in Standard and ADR-159 Fed Mice

**FIGURE 6:**
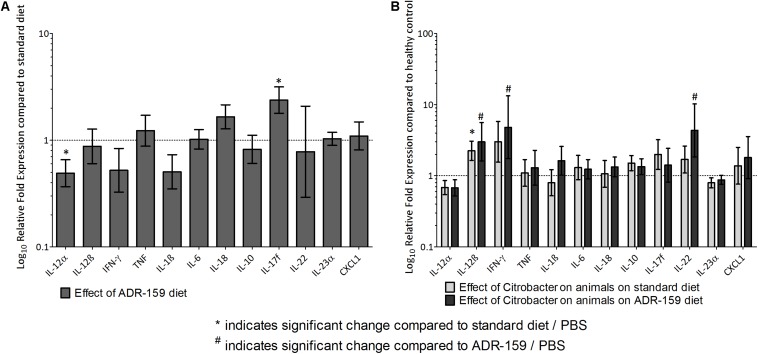
**(A)** Gene expression levels in colon at sacrifice of ADR-159 fed animals dosed with PBS compared to standard fed animals (gray). Error bars represent 95% confidence interval. Value 1 corresponds to no change in expression. *Indicates significant change compared to standard diet/PBS, # indicates significant change compared to ADR-159/PBS. **(B)** Gene expression levels in colon at sacrifice of standard (light gray) and ADR-159 (dark gray) fed animals dosed with *Citrobacter* compared to their PBS dosed counterparts. Error bars represent 95% confidence interval. Value 1 corresponds to no change in expression. ^∗^Indicates significant change compared to standard diet/PBS, ^#^indicates significant change compared to ADR-159/PBS.

Colons of ADR-159 fed animals had 0.49 times lower expression levels of IL-12α (ANOVA, *p* < 0.0005) compared to the colons of the standard fed animals. In contrast IL-17f (ANOVA, *p* = 0.0038) expression levels in colons of ADR-159 fed animals were 2.38 times higher (Figure 6). No significant differences were observed for IL-12β, IFN-γ, TNF, IL-1β, IL-6, IL-18, IL-10, IL-22, IL-23α, and CXCL1 expression levels.

Expression levels in the colons of animals dosed with *Citrobacter* were expressed relative to PBS-dosed animals on the same diet. *Citrobacter* dosing in Standard fed animals led to a significant increase in IL-12β expression. Similarly, in ADR-159 fed animals, *Citrobacter* dosing also led to a significant increase in IL-12β expression, but with additional increases in IFN-γ and IL-22 (Figure 6). There was no significant difference in IL-12α, TNF, IL-17f, IL-1β, IL-6, IL-18, IL-10, IL-23α, or CXCL1 expression level in the colons of animals dosed with *Citrobacter* on either of the diets.

### Cecum SCFA Levels in Standard and ADR-159 Fed Mice

**FIGURE 7:**
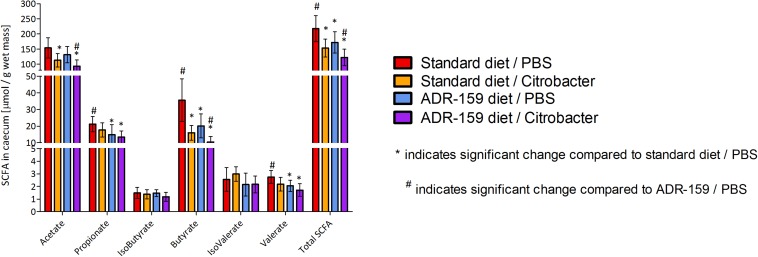
SCFA concentration in cecum of standard (red, orange) and ADR-159 (blue, purple) fed animals dosed with PBS (red, blue) or *C*. *rodentium* (orange, purple). Error bars represent SD. *Indicates significant change compared to standard diet/PBS, ^#^indicates significant change compared to ADR-159/PBS.

Total SCFA levels in the cecum differed between groups [one-way ANOVA; *F*(3, 42) = 15.489, *p* < 0.0005; Figure 7]. In particular, ADR-159 fed animals had lower levels of propionate (*post hoc* Bonferroni, *p* = 0.017), butyrate (*post hoc* Games–Howell, *p* = 0.015) and valerate (*post hoc* Bonferroni, *p* = 0.014) compared to standard fed animals. There was no effect of test conditions on levels of isobutyrate [one-way ANOVA; *F*(3, 42) = 1.797, *p* = 0.162) and isovalerate [one-way ANOVA; *F*(3, 42) = 2.988, *p* = 0.042; *post hoc* Bonferroni, *p* > 0.05]. Overall, *Citrobacter* dosed animals had lower levels of acetate, butyrate and total SCFA as compared to their uninfected counterparts.

## Discussion

The objectives of this study were to evaluate the effects of ADR-159 on the murine microbiome and on the susceptibility of mice to infection with the enteric pathogen *C. rodentium* and more specifically the impact on pathogen-induced inflammation and physiological changes associated with colitis. Female mice were fed standard chow or chow supplemented with 5% ADR-159, a heat-treated co-fermentate of *L*. *fermentum* and *L*. *delbrueckii*. After 4 weeks of these diets, animals were dosed with either *Citrobacter* or PBS to evaluate the potential protective effect of ADR-159 on infection and pathogen-induced inflammatory damage.

Prolonged consumption of high doses of ADR-159 did not cause abnormalities in animal weight, length of intestines or weight of internal organs. The ADR-159 diet did not increase fecal LCN2 levels, which is used as a proxy for intestinal inflammation. The expression levels of the majority of the cytokines in the colon was not affected by ADR-159, but we did detect a significant increase in the expression of IL-17f (see below) and a reduced expression of IL-12α (p35; see below) in ADR-159 fed animals.

The *C. rodentium* infection model is a well-established murine model which has been used previously to study bacterial pathogenesis, the health benefits of probiotics, mucosal immunology, and the role of the microbiome during infection (Crepin et al., 2016). Infection of mice with *C*. *rodentium* results in colonization of the large intestine and a subsequent mild inflammation that is maintained after the clearance of the pathogen (Wiles et al., 2004): consequently, the model is viewed as one that is relevant to IBD. During the *Citrobacter* infection phase we observed no effect of the ADR-159 diet on shedding of *Citrobacter* cells, suggesting there was no effect of ADR-159 on prevention of infection or the infection process: however, it should be noted that the high heterogeneity in initial infection observed in both the standard and ADR-159 fed animals obscures analysis.

### Effect of ADR-159 on Microbiome

The microbiome of ADR-159 fed animals differed significantly from standard fed animals based on 16S rDNA analysis. During the initial 4 weeks, the ADR-159 fed animals demonstrated a stable reduction of *Turicibacter* and *Clostridium sensu stricto*. *Turicibacter* has been associated with 2,4,6-trinitrobenzene sulfonic acid (TNBS)-induced colitis in mouse models (Jones-Hall et al., 2015) and with obesity/diabetes in mice (Horie et al., 2017). Members of *Clostridium sensu stricto* include, among others, pathogenic *Clostridium botulinum*, *Clostridium perfringens*, and *Clostridium tetani* (Gupta and Gao, 2009). The extent of the observed diet-induced microbiome shift was comparable to the previously observed change in male mice (Warda et al., 2018). However, the taxa affected in female mice differed compared to male mice (increase of *Prevotella*, reduction of *Alistipes*, and *Odoribacter*) possibly due to sex related microbiota differences.

Considering the limitations associated with utilization of 16S rDNA (Poretsky et al., 2014) we aimed to improve the resolution of our analysis by studying the virome. The virome is comprised of both DNA and RNA viruses within a particular ecosystem including bacteriophage, viruses which target specific strains of bacteria. Thus, an analysis of the virome has the potential to provide better resolution than 16S rDNA as bacteriophage levels may be indicative of the host strain fluctuations. The virome analysis further corroborated the subtle, but significant, impact of the ADR-159 diet on the microbiome.

Following the *Citrobacter* dosing, *C*. *rodentium* was detected in the microbiome of all animals dosed with the bacterium and, over time, the *C*. *rodentium* levels reduced. However, we did not observe a major disruption of the microbiome due to the infection at any of the time points. Nevertheless, we observed changes in the microbiota composition of ADR-159 fed animals dosed with *Citrobacter*: increased levels of *Allobaculum* and reduced levels of *Bacteroides*, *Alistipes* and unclassified *Prevotellaceae*, as compared to uninfected controls. Increased levels of *Allobaculum* have also been reported in mice receiving a probiotic mix (*Lactobacillus acidophilus*, *Lactobacillus rhamnosus*, and *Bifidobacterium bifidum*); this was associated with reduced colitis and tumor number after azoxymethane/DSS induction (Mendes et al., 2018). *C*. *rodentium* infection was previously shown to disrupt the intestinal microbiome (Lupp et al., 2007; Hoffmann et al., 2009). *Citrobacter* infection has been reported to trigger overgrowth of *Enterobacteriaceae* (most likely due to the fact that *Citrobacter* is a member of this group) (Lupp et al., 2007). Alternatively, *Citrobacter* infection increased the abundance of *Proteobacteria*, *Clostridia*, and *Deferribacteres*, and reduced *Lactobacillaceae* (Hoffmann et al., 2009). In our study, we did not observe an increase in *Enterobacteriaceae* (except the expected increase in *C*. *rodentium*), however, the diet-induced changes in the microbiome were maintained throughout the infection period.

### Effect of ADR-159 on *Citrobacter*-Induced Colitis

We observed a marked reduction in physiological indicators of inflammation in *Citrobacter* dosed animals fed ADR-159. In comparison to standard fed animals, ADR-159 fed animals did not show a reduction of small intestine length or an increase of colon crypt length; these parameters are established indicators of gut inflammation in response to stressors (Kim et al., 2010). In line with the prevention of these morphological responses to *Citrobacter* in ADR-159 animals, at 12 days post-initial dosing we did not observe an increase in the levels of fecal LCN2, as we did in standard fed animals. However, at sacrifice both ADR-159 and standard-fed animals had elevated levels of fecal LCN2. LCN2 is commonly used as a sensitive inflammation indicator (Chassaing et al., 2012), yet it has been shown that LCN2 itself has a role in prevention of intestinal inflammation by enhancing pathogen clearance in macrophages (Toyonaga et al., 2016). Nonetheless, these findings suggest that localized *Citrobacter*-induced inflammation is lower in ADR-159 fed animals and has potentially a later onset (alternatively LCN2 response is delayed due to the lower initial inflammation), compared to standard fed animals.

In standard-fed animals at 18 days post-dosing with *Citrobacter*, only IL-12β (p40 subunit) was upregulated compared to PBS-dosed animals. However, in ADR-159 fed animals dosed with *Citrobacter* we also observed an increase of IFN-γ and IL-22 (discussed below) compared to PBS-dosed ADR-159 fed animals. IL-12β deficient mice have been shown to be more sensitive to *Citrobacter* infection and also had increased colonic pathology (Simmons et al., 2002). IL-12β is a p40 subunit of heterodimeric IL-12 [heterodimer of p40 and p35 (IL-12α)] and a subunit of IL-23 [heterodimer of p40 and p19 (IL-23α)]. IL-12 production represents a powerful pro-inflammatory response to pathogenic microorganisms particularly, as it can induce NK cells to secrete IL-22 (Zenewicz and Flavell, 2011), and it is known to have a role in conferring resistance to *C*. *rodentium* (Ngo et al., 2018). IL-12 was also shown to trigger initiation of colitis while IL-23 drives the chronic disease phase (Eftychi et al., 2019) and it is one of the most potent inducers of IL-22. As IL-12β is up to 1000 times higher expressed than the IL-12-complementing IL-12α subunit, free p40 or p40 homodimers are commonly encountered (Zundler and Neurath, 2015; Behzadi et al., 2016). In particular, p40 homodimers have been shown to antagonize IL-12 activity *in vitro* and serve as a chemoattractant for macrophages and dendritic cell (DC) (Zundler and Neurath, 2015; Behzadi et al., 2016). We speculate that as levels of IL-12α and IL-23α were not affected by *Citrobacter* dosing regardless of the diet, the elevated levels of IL-12β would most likely lead to elevated levels of p40 homodimers.

The expression of IL-22 was induced in *Citrobacter*-dosed animals on the ADR-159 diet, but not on the standard diet (Figure 6). IL-22 belongs to the IL-10 cytokine family (Zenewicz and Flavell, 2011) and is produced in the intestinal mucosa by group 3 innate lymphoid cells (ILC3s; produces IL-22 during the early stage of infection) and CD4 + T cells (including Th17 cells and Th22 cells; produces IL-22 during late stage of infection) (Ngo et al., 2018). *C*. *rodentium* infection is known to trigger the expression of IL-22, which is then able to confer resistance to *C*. *rodentium* by binding to IL-22R, expressed by intestinal epithelial cells, and a resultant induction of mucin production. In addition, this binding also induces RegIII antimicrobial peptides, epithelial proliferation, and wound healing and repair mechanisms. The overall effect is a strengthening of barrier integrity (Ngo et al., 2018). The IL-22 response to *C*. *rodentium* infection seems to be dependent on IL-23 and a lymphotoxin pathway (Collins et al., 2014b). However, while we observed increased relative expression of IL-23 p40 (IL-12β) subunit and no change in p19 (IL-23α) subunit; we cannot conclude on the IL-23 levels. Moreover, we did not observe increased expression of CXCL1, one of the chemokines known to recruit neutrophils to the lamina propria soon after infection (Koroleva et al., 2015), in *Citrobacter* dosed animals regardless of the diet, suggesting that IL-22 was upregulated via a different pathway.

Sample collection time could be one reason for the reduced number of differentially expressed cytokines, as a number of studies have reported that the peak response (IL-17a, IL-17f, IL-22, IL-1β, TNF, and IFN-γ) is between 8 and 21 (Lupp et al., 2007; Hoffmann et al., 2009; Mondelaers et al., 2016) days post-dosing.

In PBS-dosed ADR-159 fed animals, we observed a reduced expression of IL-12α. IL-12α (p35) is a subunit of a pleiotropic cytokine IL-12 (see above) and at the same time a subunit of IL-35 (together with IL-27β), an immune-suppressing cytokine that has a role in inhibition of arthritis, asthma, and IBD (Behzadi et al., 2016; Bello et al., 2018). In view of these considerations, a reduced expression of IL- 12α in PBS-dosed ADR-159 fed animals could lead to dual effects, as both pro- and anti-inflammatory effects can be anticipated. Relevant to this is the fact that in PBS-dosed ADR-159 fed animals we did not detect altered levels of LCN2, which is a proxy for (sub-)colitis inflammation (Chassaing et al., 2012); indicating that we did not observe a pro-inflammatory effect in PBS-dosed ADR-159 fed animals.

We observed elevated levels of IL-17f in PBS-dosed ADR-159 fed animals. Although IL-17f is highly homologous to IL-17a and both of those cytokines bind to the same receptor, their functional roles differ (Ishigame et al., 2009). The primary function of IL-17f is in neutrophil recruitment and immunity to extracellular pathogens (bacterial and fungal), while IL-17a has a predominant role in autoimmune pathology (Gaffen, 2009; Ishigame et al., 2009; Tang et al., 2018). Protection against *C*. *rodentium* is known to require IL-17a and IL-17f (Ishigame et al., 2009; Collins et al., 2014b), however, IL-17f-deficient mice are resistant to dextran sodium sulfate (DSS)-induced colitis, presenting milder acute intestinal inflammation (Leppkes et al., 2009; Tang et al., 2018). Elevated levels of IL-17f in PBS-dosed ADR-159 fed animals may have the potential to enhance future resistance to pathogens: however, we did not observe reduced colonization/shedding of *Citrobacter*. It is possible that IL-17f levels may have not been elevated prior dosing or the levels reached were not at physiologically significant level. Moreover, the dual role of IL-17 may explain the lack of altered levels of IL-17f following the *Citrobacter* dosing regardless of the diet. Similarly, expression levels of IL-18, a pro-inflammatory cytokine involved in Th1 and Th2 responses and induction of cell-mediated immunity following exposure to microbial products including lipopolysaccharide (LPS) (Kaplanski, 2018), were unaffected by diet or by *Citrobacter* infection. Likewise, levels of IL-10, an essential anti-inflammatory cytokine reported to have important role as a negative regulator of immune responses to microbial antigens (Rutz and Ouyang, 2016), were not affected.

A number of pre-clinical and clinical interventions using prebiotics (Wilson and Whelan, 2017), probiotics (live bacteria) (Thakur et al., 2016) as well as dead bacteria (Lin et al., 2007; Taverniti and Guglielmetti, 2011) have been shown to have an effect on infection and/or inflammation. In line with our observations galactooligosaccharide (GOS) supplementation was shown to reduce the *Citrobacter*-induced inflammation without affecting the initial infection (Kittana et al., 2018). Similarly, rectal dosing with 140 mM butyrate did not affect *Citrobacter* infection but did reduce colon histopathological scores and hyperplasia (Jiminez et al., 2017). In our study ADR-159 fed animals had lower levels of total SCFA, in particular butyrate, propionate, and valerate. *Citrobacter* dosed animals had lower levels of acetate, butyrate and total SCFA regardless of the diet. SCFA production can contribute directly to host energy metabolism with acetate and propionate being absorbed and metabolized by the liver and peripheral organs (den Besten et al., 2013). While butyrate is mainly utilized by the colonic epithelium (den Besten et al., 2013; LeBlanc et al., 2017) and it can also be used by certain bacteria as an energy source. We did not observe an increase in chow consumption or changes in weight compared to standard fed animals, suggesting that the detected change in SCFA levels did not disturb energy levels. A number of studies have also reported SCFA levels that were far lower than those observed here as normal for C57 mice (Wang et al., 2016; Burokas et al., 2017). Although we observed a statistically significant change in SCFA in ADR-159 fed animals, this may not be physiologically relevant in a situation where there is no deficiency as was the case in our study. This is also supported by the overall positive effect that we saw in the gut in response to *Citrobacte*r challenge.

It has previously been demonstrated that *Citrobacter*-induced crypt hyperplasia was reduced by daily treatment of mice with fermented dairy products containing *Lactobacillus rhamnosus* and two strains of *Lactobacillus paracasei*, yet this treatment did not affect *C*. *rodentium* colonization (Collins et al., 2014a). Interestingly, protection against *Citrobacter*-induced crypt hyperplasia required the presence of live bacteria (Collins et al., 2014a). Collins and colleagues (Collins et al., 2014a) associated this protective effect with preventing a reduction in the abundance of *Turicibacter* spp. and *Ruminococcus* spp. at the highest point of the *C*. *rodentium* infection (Collins et al., 2014a). Loss of both *Turicibacter* spp. and *Ruminococcus* spp. was previously associated with a sensitivity to dextran sodium sulfate (DSS)-induced colitis in mice (Zenewicz et al., 2013). In the current study, ADR-159 diet alone caused consistent reduction in *Turicibacter* spp. but not *Ruminococcus* spp., this was maintained after *C*. *rodentium* dosing. This indicates that reduction in a single taxa was not associated with hyperplasia and/or colitis, as uninfected animals had no observed signs of inflammation.

## Conclusion

In conclusion, prolonged consumption of heat-treated lactobacilli (ADR-159) diet had no negative effect on general health. ADR-159 diet has an effect on microbiota composition and diversity in both male (Warda et al., 2018) and female (this study) mice. *C*. *rodentium* infection of female mice was not prevented by the ADR-159 diet: however, *Citrobacter*-induced inflammatory damage due to colitis was reduced. Simultaneously with the reduced *Citrobacter*-induced inflammation we saw induced expression of IL-12β, IFN-γ, and IL-22, known to be involved in *Citrobacter* resistance (Ngo et al., 2018).

Our studies add to the existing body of knowledge regarding the impact of heat-treated microbes on the microbiome and on health. The limited number of studies in this field suggests a need for further research. The microbiological, histological, immunological and biochemical results outlined here provide new insights on the impact of heat-treated bacteria and their metabolites on the murine microbiome and health.

These results also indicate that further investigation of the potential therapeutic effects of ADR-159 in IBD are warranted.

## Data Availability Statement

The datasets generated for this study can be found in the Sequence Read Archive under the accession PRJNA545247.

## Ethics Statement

The animal study was reviewed and approved by the Animal Experimentation Ethics Committee of University College Cork and Health Products Regulatory Authority under HPRA Project License AE19130/P060.

## Author Contributions

AW and CH contributed to the conception and design of the study. CMH and AW performed the animals experiment. AW, PB, CMH, and GD processed the samples. AC performed the analysis of microbiome and virome sequencing data. AW performed the statistical analysis and wrote the first draft of the manuscript. CMH and AC wrote sections of the manuscript. All authors contributed to manuscript revision, read, and approved the submitted version.

## Conflict of Interest

Adare Pharmaceutical provided funding for the study. Adare Pharmaceuticals was involved in study design, and in the decision to submit the article for publication, but not in the collection, analysis and interpretation of data.

## Footnotes

1 www.myassays.com
