# Supplementary material for: Oral Administration of Heat-Treated Lactobacilli Modifies the Murine Microbiome and Reduces Citrobacter Induced Colitis
Source: Front Microbiol. 2020 Jan 30;11:69. doi: 10.3389/fmicb.2020.00069 (PMC7003559; doi:10.3389/fmicb.2020.00069)
Supplement: Supplementary file 1 [file Data_Sheet_1.docx]

Suplementary material

n=12

n=12

n=12

n=12

**Table S1.** Primers used in the study

| **Target** | | **Probe** | **Primer F or R** | **Sequence** | **Product Length** | **TM** | **GC%** | **Intron spanning** | **Amplicon** | **Target used for primer design** |
| --- | --- | --- | --- | --- | --- | --- | --- | --- | --- | --- |
|  |  |  |  |  |  |  |  |  |  |  |
| Target | IFN-γ | 21 | F | atctggaggaactggcaaaa | 89 | 59 | 45 | Yes | atctggaggaactggcaaaaggatggtgacatgaaaatcctgcagagccagattatctct ttctacctcagactctttgaagtcttgaa | NM_008337.4 Mus musculus interferon gamma (Ifng), mRNA |
|  |  |  | R | ttcaagacttcaaagagtctgagg |  | 59 | 42 |  |  |  |
| Target | IL-1β | 38 | F | agttgacggaccccaaaag | 75 | 59 | 53 | Yes | agttgacggaccccaaaagatgaagggctgcttccaaacctttgacctgggctgtcctga tgagagcatccagct | NM_008361.4 Mus musculus interleukin 1 beta (Il1b), mRNA |
|  |  |  | R | agctggatgctctcatcagg |  | 60 | 55 |  |  |  |
| Target | Il 6 | 6 | F | gctaccaaactggatataatcagga | 78 | 59 | 40 | Yes | gctaccaaactggatataatcaggaaatttgcctattgaaaatttcctctggtcttctgg agtaccatagctacctgg | NM_031168.2 Mus musculus interleukin 6 (Il6), transcript variant 1, mRNA |
|  |  |  | R | ccaggtagctatggtactccagaa |  | 60 | 50 |  |  |  |
| Target | IL-10 | 41 | F | cagagccacatgctcctaga | 79 | 59 | 55 | Yes | cagagccacatgctcctagagctgcggactgccttcagccaggtgaagactttctttcaa acaaaggaccagctggaca | NM_010548.2 Mus musculus interleukin 10 (Il10), mRNA |
|  |  |  | R | tgtccagctggtcctttgtt |  | 60 | 50 |  |  |  |
| Target | IL-23 alpha subunit (p19) | 19 | F | tccctactaggactcagccaac | 78 | 59 | 55 | Yes | tccctactaggactcagccaactcctccagccagaggatcacccccgggagacccaacag atgcccagcctgagttct | NM_031252.2 Mus musculus interleukin 23, alpha subunit p19 (Il23a), mRNA |
|  |  |  | R | agaactcaggctgggcatc |  | 60 | 58 |  |  |  |
| Target | IL-17f | 46 | F | cccaggaagacatacttagaagaaa | 113 | 59 | 40 | Yes | cccaggaagacatacttagaagaaagtacttcctgagggaagaagcagccattggagaaa ccagcatgaagtgcacccgtgaaacagccatggtcaagtctttgctactgttg | ENSMUST00000039046.9\|ENSMUSG00000041872.9 Il17f-001 interleukin 17F |
|  |  |  | R | caacagtagcaaagacttgacca |  | 59 | 43 |  |  |  |
| Target | IL-18 | 46 | F | caaaccttccaaatcacttcct | 78 | 59 | 41 | Yes | caaaccttccaaatcacttcctcttggcccaggaacaatggctgccatgtcagaagactcttgcgtcaacttcaagga | NM_008360.1 Mus musculus interleukin 18 (Il18), mRNA |
|  |  |  | R | tccttgaagttgacgcaaga |  | 59 | 45 |  |  |  |
| Target | IL-12β | 82 | F6 | gcgcaagaaagaaaagatgaa | 93 | 59 | 38 | Yes | gcgcaagaaagaaaagatgaaggagacagaggaggggtgtaaccagaaaggtgcgttcctcgtagagaagacatctaccgaagtccaatgcaa | ENSMUST00000170513.2\|ENSMUSG00000004296.14 Il12b-201 interleukin 12b |
|  |  |  | R6 | ttgcattggacttcggtaga |  | 59 | 45 |  |  |  |
| Target | IL-12a | 27 | F2 | gtttaccactggaactacacaagaac | 113 | 59 | 42 | Yes | gtttaccactggaactacacaagaacgagagttgcctggctactagagagacttcttccacaacaagagggagctgcctgcccccacagaagacgtctttgatgatgaccctg | ENSMUST00000107816.3\|ENSMUSG00000027776.12 Il12a-001 interleukin 12a |
|  |  |  | R2 | cagggtcatcatcaaagacg |  | 59 | 50 |  |  |  |
| Target | IL-22 | 94 | F | tgacgaccagaacatccaga | 85 | 60 | 50 | Yes | tgacgaccagaacatccagaagaatgtcagaaggctgaaggagacagtgaaaaagcttggagagagtggagagatcaaggcgatt | NM_016971.2 Mus musculus interleukin 22 (Il22), mRNA |
|  |  |  | R | aatcgccttgatctctccac |  | 59 | 50 |  |  |  |
| Target | CXCL1 | 83 | F | acactccaacacagcaccat | 119 | 59 | 50 | Yes | acactccaacacagcaccatgatcccagccacccgctcgcttctctgtgcagcgctgctgctgctggccaccagccgcctggccacaggggcgcctatcgccaatgagctgcgctgtca | ENSMUST00000031327.8\|ENSMUSG00000029380.11 Cxcl1-001 chemokine (C-X-C motif) ligand 1 |
|  |  |  | R | tgacagcgcagctcattg |  | 60 | 56 |  |  |  |
| Target | TNF-α | 78 | F5 | cagcaaaccaccaagtgga | 82 | 60 | 53 | No | cagcaaaccaccaagtggaggagcagctggagtggctgagccagcgcgccaacgccctcctggccaacggcatggatctcaa | D84196.2\|D84196:EMBL\|60d24f1464fa67e1d8eaa780e057b0f3:MD5\|ENSMUSG00000024401:Ensembl- Gn\|MGP_129S1SvImJ_G0023628:Ensembl-Gn\|MGP_AJ_G0023587:Ensembl-Gn\|MGP_AKRJ_G0023556:Ensembl- Gn\|MGP_BALBcJ_G0023594:Ensembl-Gn\|MGP_C3HHeJ_G0023355:Ensembl-Gn\|MGP_C57BL6NJ_G0024033:Ensembl- Gn\|MGP_CASTEiJ_G0022851:Ensembl-Gn\|MGP_CBAJ_G0023333:Ensembl-Gn\|MGP_DBA2J_G0023461:Ensembl- Gn\|MGP_FVBNJ_G0023428:Ensembl-Gn\|MGP_LPJ_G0023538:Ensembl-Gn\|MGP_NODShiLtJ_G0023450:EnsemblGn\|MGP_NZOHlLtJ_G0024078:Ensembl-Gn\|MGP_PWKPhJ_G0022599:Ensembl-Gn\|MGP_WSBEiJ_G0022915:Ensembl- Gn\|ENSMUST00000025263:Ensembl-Tr\|MGP_129S1SvImJ_T0047387:Ensembl-Tr\|MGP_AJ_T0047375:Ensembl- Tr\|MGP_AKRJ_T0047324:Ensembl-Tr\|MGP_BALBcJ_T0047333:Ensembl-Tr\|MGP_C3HHeJ_T0047060:Ensembl- Tr\|MGP_C57BL6NJ_T0047808:Ensembl-Tr\|MGP_CASTEiJ_T0047142:Ensembl-Tr\|MGP_CBAJ_T0046995:Ensembl- Tr\|MGP_DBA2J_T0047107:Ensembl-Tr\|MGP_FVBNJ_T0047068:Ensembl-Tr\|MGP_LPJ_T0047211:Ensembl- Tr\|MGP_NODShiLtJ_T0047066:Ensembl-Tr\|MGP_NZOHlLtJ_T0047909:Ensembl-Tr\|MGP_PWKPhJ_T0046732:Ensembl- Tr\|MGP_WSBEiJ_T0046403:Ensembl- Tr\|PMC1061546:EuropePMC\|PMC148819:EuropePMC\|PMC434242:EuropePMC\|PMC517445:EuropePMC\|PMC523004:EuropePM C\|PMC538991:EuropePMC Mus musculus TNFA gene for tumor necrosis factor alpha, complete cds. |
|  |  |  | R5 | ttgagatccatgccgttg |  | 59 | 50 |  |  |  |
| Potential norm. gene | Tbp (TATA box binding protein, NM_013684.3 | 107 | F | ggcggtttggctaggttt | 86 | 60 | 56 | Yes | ggcggtttggctaggtttctgcggtcgcgtcattttctccgcagtgcccagcatcactatttcatggtgtgtgaagataacccaga | ENSMUST00000118001.7\|ENSMUSG00000014767.16 Tbp-002 TATA box binding protein |
|  |  |  | R | tctgggttatcttcacacacca |  | 60 | 45 |  |  |  |
| Potential norm. gene | GAPDH (glyceraldehyde-3-phosphate dehydrogenase NM_008084.3) | 33 | F | aagagggatgctgcccttac | 89 | 60 | 55 | Yes | aagagggatgctgcccttaccccggggtcccagcttaggttcatcaggtaaactcaggagagtgtttcctcgtcccgtagacaaaatgg | NM_001289726.1 Mus musculus glyceraldehyde-3-phosphate dehydrogenase (Gapdh), transcript variant 1, mRNA |
|  |  |  | R | ccattttgtctacgggacga |  | 60 | 50 |  |  |  |

**Figure S1.** (A) Average *C*. *rodentium* levels shed by standard (purple) and ADR-159 (orange) fed animals dosed with *C*. *rodentium*. Average *C*. *rodentium* levels shed by standard (blue) and ADR-159 (red) fed animals dosed with PBS. Error bars represent SD; (B) infection proportions for *C*. *rodentium* dosed animals on standard (circle) or ADR-159 (square) diet (C) shedding of individual *C*. *rodentium* infected animals on standard diet; (D) shedding of individual *C*. *rodentium* infected animals on ADR-159 diet.


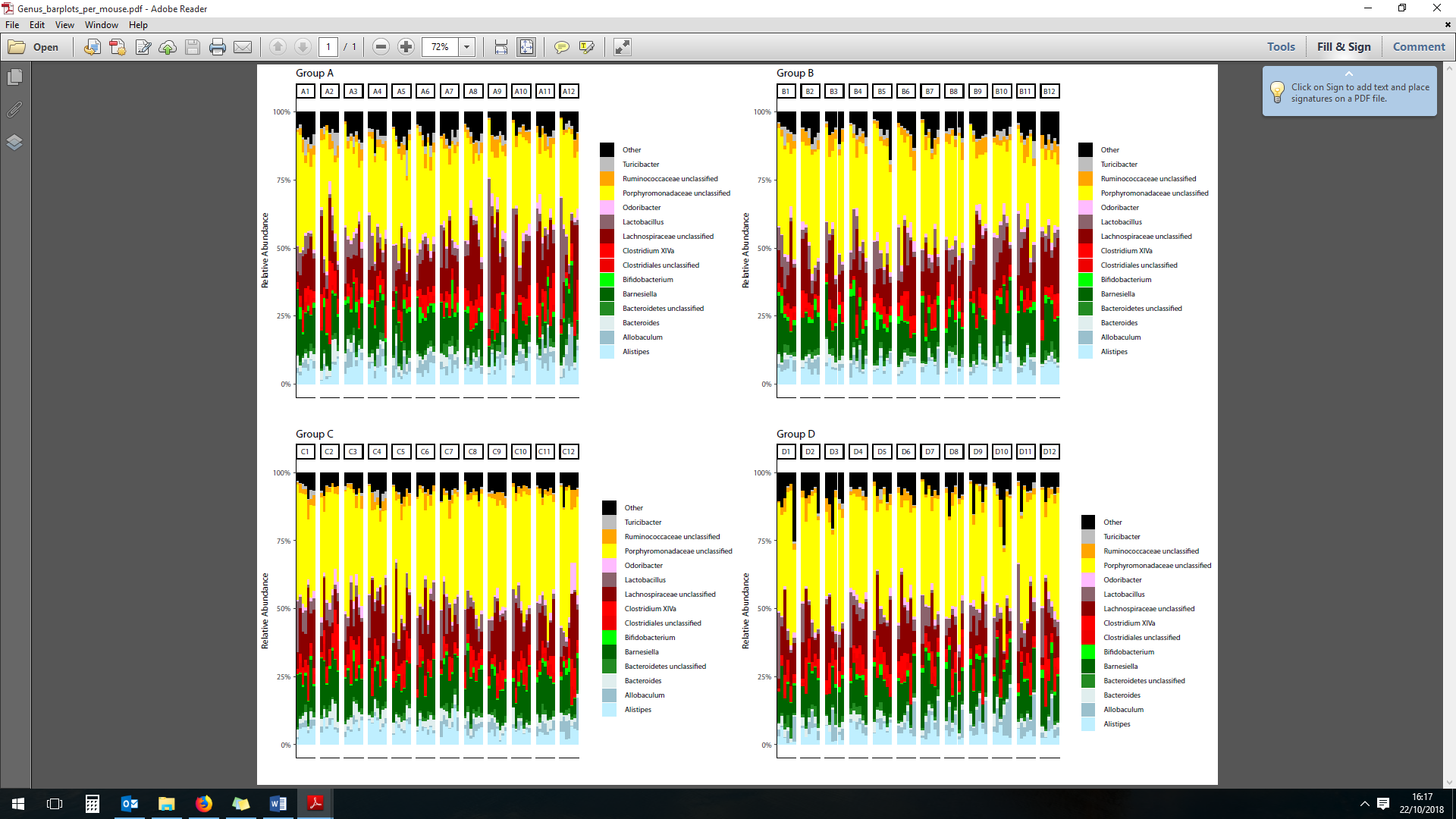


**Figure S2.** Relative abundance at genus level throughout the whole experiment for individual standard (A, B) and ADR-159 (C, D) fed animals dosed with *C*. *rodentium* (B, D) or PBS (A, C). Individual animals labelled 1 to 12. For each animal from left to right: week 0, week 2, week 3 (only groups A&C), week 4 (before dosing), 8 days post dosing, 12 days post dosing and 18 days post dosing (sacrifice).

**Table S2.** RSV differently abundant in ADR-159 fed animals compared to standard fed ones. Red indicated higher abundance; green indicates lower abundance in ADR-159 fed animals. For clarification genus and species assign to given RSV are presented.

**Table S3.** Supplementary genus level comparisons post dosing with PBS or *Citrobacter*. * indicated taxa significantly different at one of the initial 4 weeks of experiment.

**Table S3.** RSV differently abundant in standard and ADR-159 fed animals before and 8, 12 and 18 days post dosing with PBS or *Citrobacter*. For clarification genus and/or species assign to given RSV are presented.

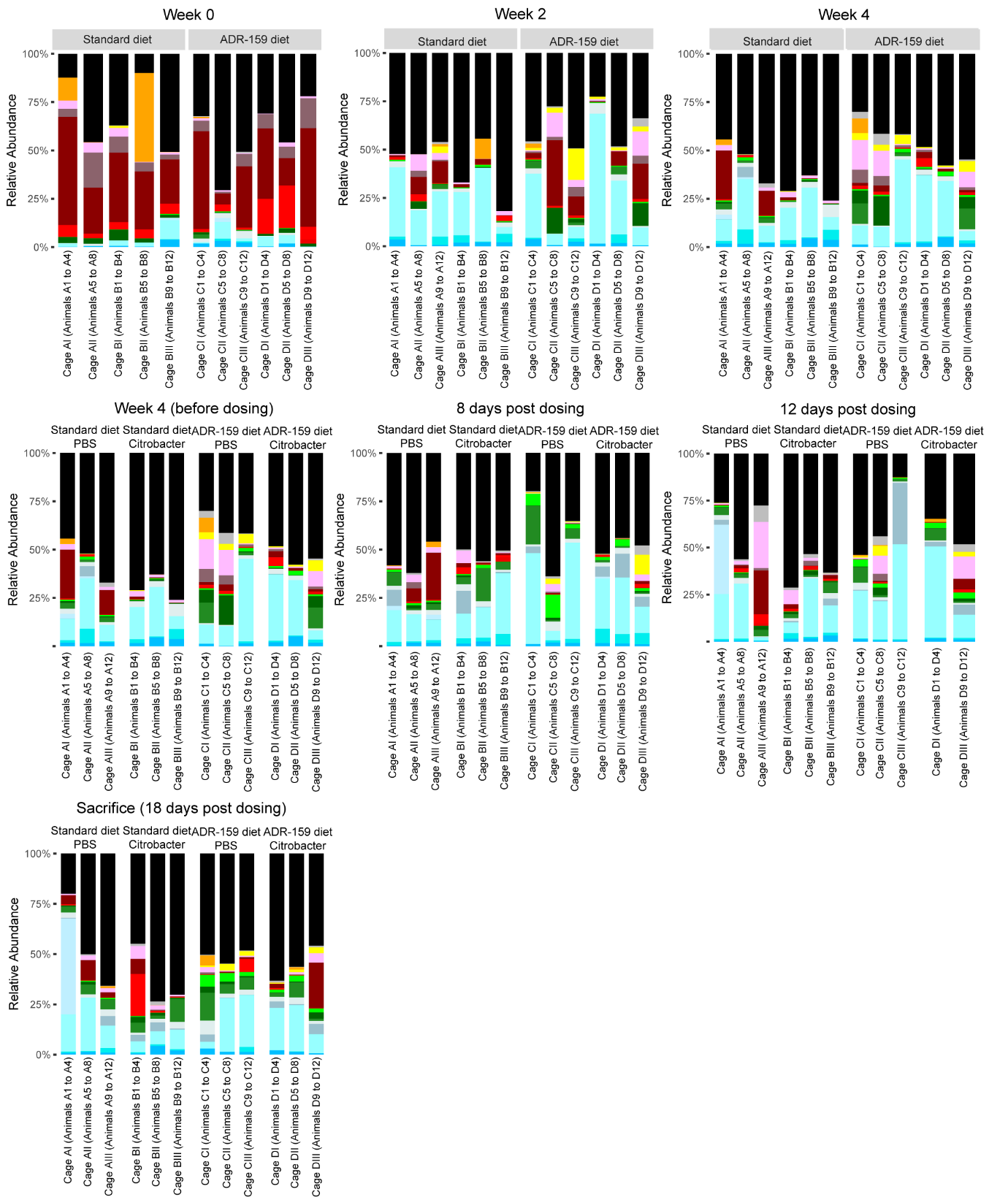


**Figure S3.** Relative abundance of viral contigs throughout the whole experiment for cages of standard (A, B) and ADR-159 (C, D) fed animals dosed with *C*. *rodentium* (B, D) or PBS (A, C). Each color represents different contig, for simplicity, contigs with overall abundances below 1% were grouped together (black).

**Table S4.** P-values for virom alpha and beta diversity before dosing

| **Time point** | **Standard vs ADR-159 diet** | |
| --- | --- | --- |
|  | **Alpha diversity** | **Beta diversity** |
| Week 0 | 0.3290 | 0.4645 |
| Week 2 | 0.0152 | 0.1618 |
| Week 4 | 0.1320 | 0.0480 |

**Table S5.** P-values for virom alpha and beta diversity after dosing
